# Bone Mineral Density During Treatment with The Janus Kinase Inhibitor Baricitinib in Patients with Rheumatoid Arthritis: A Monocentric Observational Study

**DOI:** 10.1007/s00223-025-01410-9

**Published:** 2025-07-22

**Authors:** Nils Schulz, Thomas Asendorf, Pascal van Wijnen, Tim Wilhelmi, Ulf Müller-Ladner, Uwe Lange, Philipp Klemm

**Affiliations:** 1 Department of Rheumatology, Clinical Immunology, Osteology and Physical Medicine, Justus Liebig University Giessen, Campus Kerckhoff, Benekestr. 2-8, 61231 Bad Nauheim, Germany; 2 Department of Medical Statistics, University Medical Center Göttingen, Göttingen, Germany

**Keywords:** Baricitinib, Bone mineral density, Janus kinase inhibitor, Rheumatoid arthritis

## Abstract

Rheumatoid arthritis (RA) is associated with systemic bone loss and thus an established risk factor for osteoporosis. Janus kinase inhibitors (JAKi) have shown osteo-protective effects. However, clinical data on the effects of baricitinib on bone mineral density (BMD) remain limited. Therefore, we investigated the effects of a 1-year treatment with baricitinib on BMD in RA patients. Patients with active RA beginning treatment with baricitinib were included. BMD was measured at the lumbar spine and femoral neck using Dual-Energy X-Ray Absorptiometry (DXA). Disease activity was assessed using DAS28-CRP and cDAI. The primary endpoint was the change in BMD after 12 months. Secondary endpoints evaluated changes in disease activity, prednisolone dose and alkaline phosphatase (AP) levels and its relation to BMD. A total of 46 RA patients were recruited, of whom 26 completed the study. Overall, BMD remained stable. Non-responders to baricitinib (based on DAS28-CRP) showed a significant decline in spine BMD (− 2.12%, *p* = 0.039), while responders showed stable BMD. The between-group difference in spine BMD (*p* = 0.008) and T-score (*p* = 0.012) was significant. Demographic and clinical characteristics did not differ significantly between groups. Disease activity (DAS28-CRP: *p* = 0.003; cDAI: *p* = 0.007), prednisolone dose (*p* = 0.006), and AP levels (*p* = 0.03) all improved significantly. Under baricitinib, BMD loss appeared stabilized in RA patients. Non-responders to baricitinib experienced a significant loss of BMD with a significant difference to responders raising the question if seen effects are achieved by controlling disease activity or if there is an additional explicit JAKi effect on bone metabolism. Trial registration number: DRKS00020780, date: 13.3.2020.

## Introduction

Rheumatoid arthritis (RA) is the most common inflammatory rheumatic disease in Germany, affecting approximately 0.8–1.2% (around 560,000–830,000 individuals) of the adult population [1]. In addition to pain and functional limitations due to joint and bone destruction, RA patients have an increased risk of reduced bone mineral density (BMD). Moreover, RA is an established risk factor of osteoporosis (OP). Over 20% of RA patients develop OP during the course of their disease [2]. Particularly, prolonged disease duration and cumulative glucocorticoid exposure are known risk factors for OP in this population [3].

While OP-related fractures cause billions of euros in healthcare costs annually in Europe [4], the impact of OP on an individual level can be even more deleterious. Besides pain and impairment, OP-related fractures are associated with increased mortality: Approximately 30% of deaths after hip or clinical spine fractures are directly linked to the fracture event [4].

OP results from an impairment of the physiological bone remodeling process, driven by increased bone resorption, decreased bone formation, or a combination of both [5]. Recent studies have highlighted the role of inflammatory mediators such as the pro-inflammatory cytokines interleukin-1 (IL-1), interleukin-6 (IL-6), and tumor necrosis factor-α (TNF-α) in the pathogenesis of OP [6–8]. Based on these findings, RA therapies targeting these cytokines or their signaling pathways have demonstrated an osteo-protective effect [9]. For instance, biological (b) disease-modifying antirheumatic drugs (DMARDs), such as TNF inhibitors (TNFi), tocilizumab, abatacept or rituximab, stabilize or reduce RA-associated bone loss [10].

Diving deeper into the pathogenesis of OP recent in vitro studies revealed that inhibition of Janus kinase (JAK) or Signal Transducers and Activators of Transcription (STAT), particularly through modulation of the Receptor Activator of NF-κB Ligand (RANKL) signaling pathway, suppresses osteoclastogenesis and stimulates osteoblastogenesis [11–15]. As JAK inhibitors (JAKi) have become part of routine clinical practice nowadays, these molecular effects induce novel research questions addressing the specific role of JAKi in osteometabolism [16–19].

Due to the osteo-immunological rationale and positive data regarding bDMARD therapies [10], the present study aimed to investigate the effects of a one-year treatment with baricitinib on BMD in RA patients.

## Methods

### Study Design

This study is a prospective observational cohort study including patients, who initiated treatment with the JAKi baricitinib due to active RA between 2019 and 2021. Patients were followed for 12 months after initiating therapy, with data collection at baseline and follow-up (after 12 months).

### Setting

Recruitment and data collection were conducted at the Department of Rheumatology, Clinical Immunology, Osteology, and Physical Medicine at Justus Liebig University Giessen, Campus Kerckhoff in Bad Nauheim, Germany. All patients were thoroughly informed about the study and provided written consent to participate. The study was approved by the Ethics Committee of Justus Liebig University Giessen (AZ 149/18) and prospectively registered in the German Registry of Clinical Studies (DRKS) under the number DRKS00020780.

### Participants

Patients who met the 2010 ACR/EULAR classification criteria for RA [20] and initiated baricitinib due to active RA were included in the study.

Inclusion criteria:

- Diagnosis of RA according to the 2010 ACR/EULAR criteria.
- Initiation of baricitinib therapy due to active RA.

Exclusion criteria:

- Previous use of JAKi.
- Contraindications to baricitinib therapy.
- Secondary osteoporosis unrelated to RA (e.g., glucocorticoid-induced osteoporosis).
- long-term therapy (≥ 6 months) with prednisolone at doses > 5 mg per day before inclusion
- (osteoporotic) fracture event that occurred less than 12 months ago.

Patients who discontinued baricitinib therapy during the study period were also excluded.

### Sample Size

Due to the study’s exploratory nature sample size calculation was not performed.

### Endpoints

The primary endpoint of this study was the change in BMD, assessed via DXA, after 12 months of baricitinib therapy compared to baseline.

Secondary endpoints included the therapeutic response of RA to baricitinib as measured by DAS28-CRP, cDAI, and CRP, the variation in daily prednisolone dosage after 12 months of treatment as well as changes in AP as a surrogate marker for bone metabolism. Additionally, a descriptive analysis of baricitinib drug survival and any adverse events associated with the therapy was performed.

### Variables and Measurements

#### Primary Endpoint

##### BMD

BMD measurements were performed using Dual-Energy X-Ray Absorptiometry (DXA) with a Lunar Prodigy X device (Lunar Radiation Corporation, Madison, Wisconsin, United States). Measurements were taken at the lumbar spine (L1–L4) and the femoral neck. All measurements were performed using the same device under standardized conditions. Based on internal quality assurance protocols, the least significant change (LSC) was estimated at 39 mg/cm^2^ for lumbar spine and 33 mg/cm^2^ for femoral neck.

According to WHO criteria, a decrease in BMD of > 2.5 standard deviations below the mean value for young healthy adults (T-score < − 2.5) was defined as osteoporosis. A T-score between − 2.5 and − 1 was classified as osteopenia, while a T-score > − 1 indicated age-appropriate BMD [21].

#### Secondary Endpoints

##### Disease Activity of RA

Disease activity in RA was assessed using the validated instruments Disease Activity Score 28-CRP (DAS28-CRP) and Clinical Disease Activity Index (cDAI) [22]. DAS28-CRP and cDAI scores were calculated based on clinical examination findings (+ CRP for DAS28-CRP). The DAS28 score consists of the following components: SJC 28 (swollen joint count), TJC 28 (tender joint count), PGA (patient global assessment of disease activity), and CRP. The cDAI includes SJC 28, TJC 28, PGA, and EGA (evaluator global assessment). Disease activity was categorized as follows:

- DAS28-CRP: 0–2.6 indicates remission; > 2.6–3.2 indicates low; > 3.2–5.1 indicates moderate; and > 5.1 indicates high disease activity.
- cDAI: < 2.8 indicates remission; 2.8–10 indicates low; > 10–22 indicates moderate; and > 22 indicates high disease activity (22).

Due to the observational nature of this trial, treatment was standard of care and not interventional. Therefore, treatment response to baricitinib in the analysis was defined as: a response if the DAS28-CRP at 52 weeks visit was lower than at study inclusion (DAS28-CRP endpoint < inclusion), whereas non-response was defined as a DAS28-CRP at 52 weeks that was equal to or greater than at study inclusion (DAS28-CRP endpoint ≥ inclusion).

##### Prednisolone Dosage

Daily prednisolone dosage at baseline and changes in daily prednisolone dosage were documented.

##### Laboratory Parameters

Standardized pre-analytical procedures included early morning measurement of serum calcium, 25-hydroxyvitamin D (VitD3), C-reactive protein (CRP), erythrocyte sedimentation rate (ESR), alkaline phosphatase (AP), and γ-glutamyltransferase (GGT). To exclude potential confounding factors affecting bone metabolism (such as secondary osteoporosis), thyroid-stimulating hormone (TSH) and serum creatinine were also measured.

Additionally, demographic data and potential confounders such as age and gender, as well as disease-specific data including disease duration, presence of RA-associated autoantibodies (rheumatoid factor and ACPA), number of prior bDMARD therapies, and concomitant medications were collected.

### Bias and Handling of Missing Data

To minimize bias, all data were analyzed by blinded assessors, reducing the potential for subjective influence. Confounding factors, such as secondary forms of osteoporosis were carefully excluded.

Due to the study’s exploratory and observative nature the analysis was conducted according to the per-protocol principle without the imputation of missing data.

### Data Collection

Data on disease activity and BMD were collected at baseline and after 12 months of treatment. Blood samples for laboratory markers were also collected at these time points. All data collection followed standardized protocols.

### Statistical Analysis

#### Descriptive Analysis

Continuous variables, such as BMD and disease activity scores (DAS28-CRP and cDAI), were described using the arithmetic mean and standard deviation.

The distribution of these data was tested for normality using the Shapiro–Wilk test, which informed the choice of further parametric or non-parametric tests.

Drug survival (treatment continuation) was analyzed descriptively by calculating the proportion of patients who completed the study after 12 months. Reasons for treatment discontinuation (such as inadequate response, adverse effects, and other factors) were documented.

Patients were stratified based on the number of prior bDMARD therapies to explore differences in drug survival.

#### Comparative Tests

A paired t-Test was applied to assess changes in key variables, including BMD, disease activity scores (DAS28-CRP and cDAI), daily prednisolone dosage, and other laboratory parameters from baseline to the 12-month follow-up. This test was appropriate for comparing within-subject changes over time under the assumption of normal distribution.

A unpaired T-test was used to assess changes between groups. The unpaired t-Test was used in particular to examine the difference in BMD values over time between the treatment responder and non-responder groups.

#### Analysis of Covariance (ANCOVA)

To control for confounding factors, ANCOVA was used to compare changes in BMD and corresponding T-scores between the different patient groups (treatment responders vs. non-responders). Covariates such as age, gender, disease duration, osteoporosis medication, prednisolone therapy, concurrent DMARD therapy, and vitamin D supplementation were included in this analysis to adjust for potential baseline differences.

#### Significance Level

All statistical tests were two-tailed, with a significance level set at *p* = 0.05. This alpha level was chosen to determine statistically significant changes, balancing the risk of Type I errors with study rigor.

#### Software and Blinding

Statistical analyses were performed using R version 4.4.1 for Windows, and all analyses were conducted by assessors blinded to treatment groups to prevent potential bias.

## Results

**Fig. 1:**
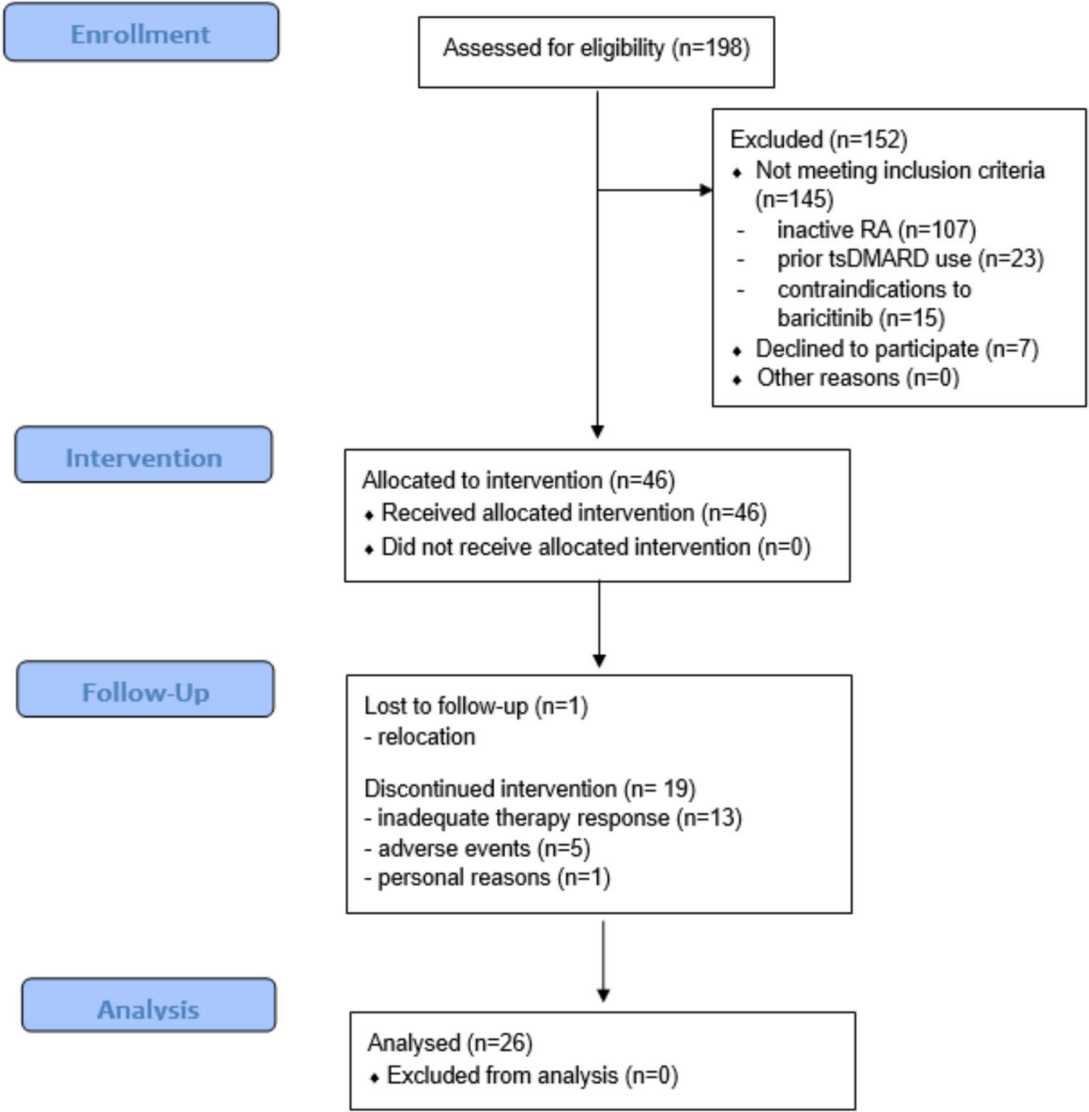
Patient recruitment and follow-up (according to STROBE guidelines)

A total of 198 patients with RA were screened for the study. Of these, 145 patients did not meet the inclusion criteria [inactive RA (*n* = 107), prior tsDMARD use (*n* = 23) or contraindications to baricitinib (*n* = 15)]. Ultimately, 46 patients were included in the study, with 26 patients completing at least 12 months of baricitinib therapy and being evaluated (Fig. 1). Table 1 presents the resulting patient characteristics.

**Table 1:** Patient characteristics at baseline

Characteristic	Inclusion	Analysis
Number of patients (n)	46	26
Gender:		
Female	33 (72%)	20 (77%)
Male	13 (28%)	6 (23%)
Age in years (mean, SD)	64 ± 11	62 ± 11
Disease duration in years (mean, SD)	10 ± 10.6	11.5 ± 10.4
Antibody status:		
RF positive	23 (50%)	11 (42%)
ACPA positive	19 (41%)	11 (42%)
Bone mineral density (WHO definition):		
Normal	11 (24%)	5 (19%)
Osteopenia	27 (59%)	15 (58%)
Osteoporosis	8 (17%)	6 (23%)
Number of previous bDMARD therapies:		
0	19 (41%)	7 (27%)
1	9 (20%)	5 (19%)
≥ 2	18 (39%)	14 (54%)
Medication:		
Baricitinib 2 mg/day	3 (7%)	3 (12%)
Baricitinib 4 mg/day	43 (93%)	23 (88%)
Additional csDMARD therapy	16 (35%)	8 (31%)
Methotrexate	15 (94%)	8 (100%)
Sulfasalazine	1 (6%)	0
Patients receiving prednisolone	33 (72%)	15 (58%)
Daily prednisolone dose (mg/day)	9.2 ± 9.5	6.6 ± 7.7
Disease activity:		
cDAI (mean, SD)	21.6 ± 8.0	21.2 ± 8.3
DAS28-CRP (mean, SD)	4.4 ± 0.9	4.4 ± 0.8
CRP mg/dl (mean, SD)	0.9 ± 1.2	0.8 ± 0.8

*RF* rheumatoid factor, *ACPA* anti-citrullinated protein antibody, *cs-/bDMARD* conventional synthetic/biological disease-modifying antirheumatic drug, *cDAI* clinical disease activity index, *DAS*28*-CRP* disease activity score 28-C-reactive protein

Of the 46 patients included at the beginning of the study, 26 patients (56.5%) completed the study. Reasons for study discontinuation were inadequate therapy response (*n* = 13, 28.3%), adverse events (*n* = 5, 10.9%), and other reasons (*n* = 2, 4.3%). Reported adverse events included herpes zoster, recurrent infections, allergic reaction, palpitations, and nausea (each *n* = 1). Cardioembolic or thrombotic events did not occur. One patient withdrew from the study for personal reasons, and another patient relocated.

At the outset of the study, six patients were administered a specific osteoporosis medication, which remained unchanged throughout the duration of the investigation.

Patients naive to bDMARDs completed the study to a lesser percentage when compared to patients with at least two prior bDMARD-therapies [*n* = 7/19 (36.8%) vs. *n* = 14/18 (77.8%)].

### BMD

Under baricitinib treatment RA patients displayed no significant changes in spine and femoral BMD measured in absolute values (g/cm^2^) as well as the T- and Z-scores over a one-year period (Table 2). Mean changes of BMD and T-scores were positive at both sites (BMD spine: Δ 8.2 mg/cm^2^ (+ 0.74%), 95% confidence interval (CI) [− 13.6;30.0], BMD femoral: Δ 7.6 mg/cm^2^ (+ 0.96%), 95% CI [− 12.6;27.8]). All changes remaining below the LSC thresholds of 39 mg/cm^2^ for the spine and 33 mg/cm^2^ for the femoral neck.

**Table 2:** Bone mineral density

Characteristic	Baseline	Endpoint	Mean change	*p* value
BMD spine (g/cm^2^)	1.12 ± 0.17	1.13 ± 0.18	0.00 ± 0.05	0.444
T-score spine	− 0.58 ± 1.40	− 0.50 ± 1.46	0.07 ± 0.43	0.401
Z-score spine	0.28 ± 1.58	0.34 ± 1.72	0.06 ± 0.45	0.499
BMD femoral neck (g/cm^2^)	0.85 ± 0.14	0.85 ± 0.15	0.01 ± 0.05	0.445
T-score femoral neck	− 1.33 ± 1.17	− 1.29 ± 1.20	0.04 ± 0.37	0.597
Z-score femoral neck	− 0.35 ± 1.20	− 0.46 ± 1.21	− 0.11 ± 0.58	0.358

The least significant change threshold for the spine BMD were 39 mg/cm^2^ and for the femoral BMD 33 mg/cm^2^

*BMD* bone mineral density

To investigate a possible JAKi/baricitinib-dependent effect on BMD, subgroup analysis was performed on treatment responders and non-responders to baricitinib based on disease activity development measured by DAS28-CRP. In the subgroup analysis, a statistically significant deterioration of spine BMD was observed within the group of non-responders to baricitinib therapy (BMD spine Δ − 25 ± 25 mg/cm^2^, 95% CI [− 49.0; − 1.8], *p* = 0.039) with a large effect size (Cohen’s *d* = − 0.996). The corresponding spine T-score did not reach statistical significance (T-score spine Δ − 0.20 ± 0.23, 95% CI [− 0.41;0.01], *p* = 0.062). There were no significant changes in femoral BMD. In the group of therapy responders, mean values of both spine and femoral BMD improved without reaching statistical significance (Table 3). While changes in BMD were positive in therapy responders (BMD spine: + 1.92%, BMD femoral: + 1.48%), all changes were negative in non-responders (BMD spine: −2.12%, BMD femoral: −0.16%). Changes in both spine BMD and the corresponding T-score differed significantly between treatment responders and non-responders (spine BMD: Δ + 22 mg/cm^2^ vs. − 25 mg/cm^2^, 95% CI [13.8; 81.2], *p* = 0.008; spine T-score: Δ + 0.19 vs. − 0.20, 95% CI [0.10;0.68], *p* = 0.012), each demonstrating a large effect size (Cohen’s d = 0.99 and 0.96, respectively), although all values remained within the LSC threshold.

**Table 3:** Subgroup analysis: BMD changes depending on changes in disease activity

Characteristic	Baseline	Endpoint	Mean change	*p* value
Responders (*n* = 18)				
BMD spine (g/cm^2^)	1.12 ± 0.17	1.14 ± 0.18	0.02 ± 0.05	0.110
T-score spine	− 0.56 ± 1.36	− 0.38 ± 1.49	0.19 ± 0.45	0.106
BMD femoral neck (g/cm^2^)	0.87 ± 0.15	0.88 ± 0.16	0.01 ± 0.06	0.371
T-score femoral neck	− 1.15 ± 1.18	− 1.08 ± 1.23	0.08 ± 0.43	0.469
Non-responders (*n* = 8)				
BMD spine (g/cm^2^)	1.11 ± 0.19	1.09 ± 0.17	− 0.03 ± 0.03	**0.039**
T-score spine	− 0.60 ± 1.60	− 0.80 ± 1.47	− 0.20 ± 0.23	0.062
BMD femoral neck (g/cm^2^)	0.80 ± 0.13	0.80 ± 0.12	− 0.00 ± 0.03	0.770
T-score femoral neck	− 1.7 ± 1.12	− 1.74 ± 1.06	− 0.04 ± 0.23	0.662

Responders and non-responders refer to the therapeutic response to baricitinib as measured by DAS28-CRP (responders DAS28-CRP endpoint < baseline, non-responders DAS28-CRP endpoint > baseline). The least significant changes for the spine BMD were 39 mg/cm^2^ and for the femoral BMD 33 mg/cm^2^

*DAS*28*-CRP* Disease Activity Score 28 C-reactive protein, *BMD* bone mineral density

The intergroup comparison revealed no significant differences in baseline characteristics (age, gender, disease duration, osteoporosis medication, prednisolone therapy, concurrent csDMARD therapy, and vitamin D supplementation), confirming that both groups were comparable in terms of their demographic and clinical parameters (all *p* > 0.05). The observed BMD changes are therefore likely attributable to treatment response (Table 3).

To assess the association between disease activity and changes in BMD, an ANCOVA was performed with change in DAS28-CRP as a covariate. For spine BMD, there was a trend toward a negative association with ΔDAS28-CRP (*p* = 0.063), suggesting that a greater improvement in disease activity may be linked to BMD stabilization. No significant association was observed for femoral neck BMD (*p* = 0.262).

Furthermore, there were no statistically significant differences in mean daily prednisolone doses (mg) between therapy responders and non-responders at baseline (6.90 vs. 6.81, *p* = 0.98) or after one year of treatment (2.34 vs. 2.00, *p* = 0.78), nor in the change in daily prednisolone dosage from baseline to endpoint between the two groups (*p* = 0.94).

**Fig. 2:**
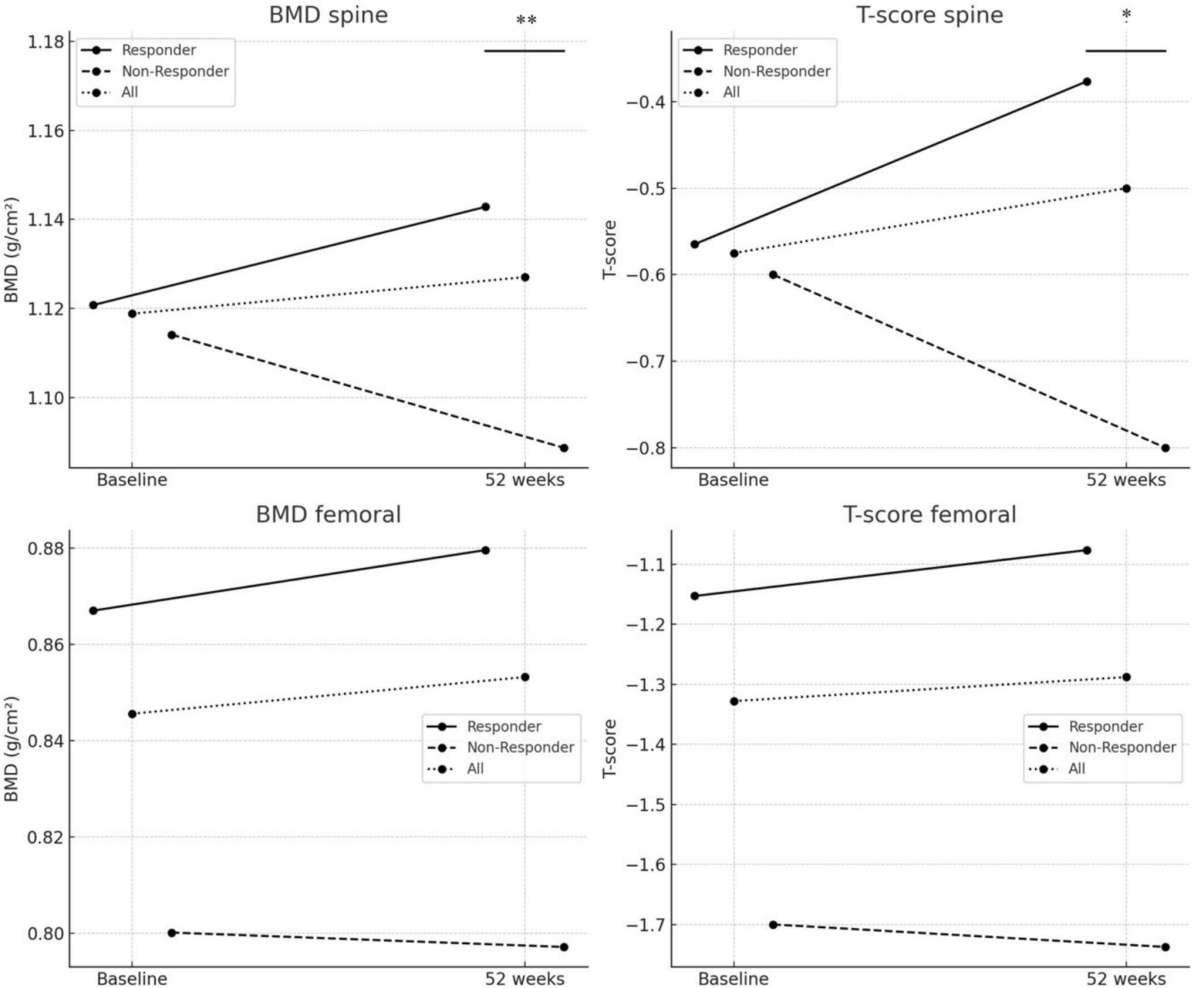
Changes of spine and femoral BMD as well as T-scores in comparison between therapy responder, non-responder and all patients over time. *p < 0.05, **p < 0.01

Changes of spine and femoral BMD as well as the T-scores are shown in Fig. 2.

### Disease activity and laboratory values

Compared to baseline, surrogate parameters for measuring disease activity in RA showed statistically significant improvements (ΔcDAI: − 6.27 ± 12.58, 95% CI [− 9.86; − 2.68], *p* = 0.007; ΔDAS28-CRP: − 0.76 ± 1.36, 95% CI [− 1.15; − 0.37], *p* = 0.003; ΔCRP: − 0.32 ± 1.04 mg/dl, 95% CI [− 0.62; − 0.02], *p* = 0.015). The average daily dose of prednisolone decreased significantly (Δ: − 4.45 ± 8.30 mg, 95% CI [− 6.82; − 2.08], *p* = 0.006). AP levels changed significantly (ΔAP: − 9.62 ± 32.86 U/l, 95% CI [− 19.00; − 0.24], *p* = 0.030), while GGT, serum calcium, and VitD3 levels did not show statistically significant changes (Table 4).

**Table 4:** Clinical and laboratory changes

Characteristic	Baseline	Endpoint	*p* value
Disease activity:			
cDAI	21.15 ± 8.25	14.88 ± 9.50	**0.007**
DAS28-CRP	4.35 ± 0.78	3.59 ± 1.12	**0.003**
Average dose of prednisolone/day (mg)	6.6 ± 7.7	2.15 ± 3.11	**0.006**
Laboratory values:			
AP U/l (Ref.: 35–104)	77.08 ± 22.44	67.46 ± 24.00	**0.030**
GGT U/l (Ref.: 6–42)	42.14 ± 41.46	53.16 ± 61.24	0.112
CRP mg/dl (Ref.: < 0.5)	0.75 ± 0.84	0.43 ± 0.61	**0.015**
Calcium mmol/l (Ref.: 2.09–2.54)	2.36 ± 0.09	2.40 ± 0.09	0.154
VitD3 ng/ml (Ref.: > 30)	30.07 ± 17.03	32.63 ± 16.45	0.584

*cDAI* clinical Disease Activity Index, *DAS*28 Disease Activity Score 28, *Ref*. reference-value, *AP* alkaline phosphatase, *GGT* γ-glutamyltransferase, *CRP* C-reactive protein, *VitD*3 25-hydroxyvitamin D

## Discussion

This study is the first to investigate the effects of baricitinib therapy on BMD (measured by DXA) in RA patients over a 12-month period.

Over the 12-month treatment period, BMD remained stable across the overall cohort, with no statistically significant changes at all sites with positive mean changes. On average, all parameters showed a slight positive trend, with spine BMD increasing by + 0.74% and femoral BMD by + 0.96%, implying that baricitinib helped to stabilize BMD in the majority of patients. This stabilization is particularly noteworthy considering that healthy individuals typically experience an average annual bone loss of about −0.2% at the spine and − 0.3 to  − 0.5% at the femur, while in patients with RA this effect is even more pronounced. RA patients experience an annual bone loss of approximately −1.75% at the spine and −1.4% at the femur resulting in a 20% higher incidence of OP compared to the general population [2, 23, 24].

To further investigate JAKi/baricitinib-dependent effects on BMD, analysis was performed clustering patients in treatment responders and non-responders to baricitinib treatment based on disease activity measured by DAS28-CRP. These subgroup analysis further supports the finding that baricitinib contributes to BMD stabilization, as non-responders to baricitinib experienced a decline in BMD, whereas responders maintained stable levels, with a statistically significant difference observed in spine BMD (therapy responders: + 1.92% vs. non-responders: −2.12%, *p* = 0.008).

It is not surprising that changes in spine BMD were particularly evident, given the well-documented evidence on BMD alterations under bone-specific therapies, in which spine BMD shows earlier and more pronounced changes compared to femoral BMD [25]. A possible explanation for this phenomenon is the higher proportion of osteo-metabolically active trabecular bone in the spine, whereas the femur consists of a greater proportion of cortical bone, which responds more slowly to metabolic changes [26].

Our results align with the BARE BONE study, which demonstrated that a 1-year treatment with baricitinib in RA patients improved both volumetric BMD of the dominant hand (measured by high-resolution peripheral quantitative computed tomography (HR-pQCT) and biomechanical properties/bone strength [27]. Similar findings have been reported for the JAKi tofacitinib, in which a 1-year treatment in comparable RA cohorts led to stabilization of both spine and peripheral BMD [28, 29].

Analogous to BMD development baricitinib demonstrated a favorable therapeutic response in the overall cohort, with significant reduction in disease activity measured by both DAS28-CRP (*p* = 0.003) and cDAI (*p* = 0.007). Additionally, the daily prednisolone dosage was significantly reduced (*p* = 0.006).

A possible mechanism for the observed decrease in BMD among non-responders could be the persistently high disease activity, which is known as a risk factor for progressive bone loss [30]. However, the multivariate ANCOVA conducted in this study showed that neither CRP nor DAS28-CRP was significantly associated with BMD in the overall cohort, although a trend toward significance was observed for change in DAS28-CRP in relation to spine BMD. This suggests that while systemic inflammation may play a role, it is unlikely to be the sole mechanism responsible for bone loss in this patient group. In fact, one can hypothesize that if one’s RA does not respond to baricitinib therapy BMD and bone metabolism will also not respond implying some sort of resistance to baricitinib treatment. The glucocorticoid dose, another known risk factor for BMD reduction [3], can also be excluded, as there were no differences between responders and non-responders in baseline, endpoint, or change in prednisolone dosage indicating that additional factors beyond inflammation and glucocorticoids are likely influencing BMD reduction in this patient group.

As demonstrated in vivo using a mouse model, the administration of JAKi resulted in senolysis, which refers to the elimination of senescent cells. This intervention was associated with increased BMD and improved bone microarchitecture, attributed to reduced bone resorption and enhanced bone formation when compared to the control group [13]. In contrast, initial in vivo studies did not demonstrate a significant difference in bone metabolism parameters following the administration of senolytic agents. However, the study utilized the tyrosine kinase inhibitor dasatinib rather than a JAKi, which complicates the comparison and may instead support the argument for the bone-protective effects of JAKi, independent of the senolytic activity [31].

It is important to note that the observed changes in BMD were controlled for vitamin D status, renal retention parameters, and thyroid function. Additionally, AP was measured, which is secreted by osteoblasts and can be used as a surrogate marker for bone formation in patients with normal liver function [32]. As demonstrated by Atalay et al., AP levels are significantly higher in osteoporosis patients and serve as a marker of pathologically increased bone turnover [33]. Furthermore, a reduction in AP has been shown to correlate with the therapeutic response to bisphosphonates [34].

In this study cohort, AP levels significantly decreased over time (*p* = 0.03) further suggesting that bone homeostasis stabilized under baricitinib therapy. Since AP [like other bone metabolism markers like procollagen type 1 N-terminal propeptide (P1NP) or C-terminal telopeptide (CTX)] correlates with RA disease activity [35, 36], this decline may reflect both improved bone homeostasis and a control of the inflammatory activity of RA, which is consistent with reductions in DAS28-CRP and cDAI scores.

Interestingly, GGT, which is also linked to RA disease activity [37], showed a numerical increase, supporting the interpretation that the AP decline is more likely bone-related than hepatic in origin.

Comparable results have been published for RA patients starting therapy with the JAKi tofacitinib: after one year of treatment, markers of bone formation significantly increased, while markers of bone resorption significantly decreased [28, 29].

26 out of 46 patients (56.5%) reached the endpoint and completed the study (with most dropouts due to lack of efficacy). This relatively high dropout rate may reflect the real-world character of the cohort, which included older and treatment-refractory patients (mean age 62 ± 11 years), and the timing of study inclusion during the early phase of the COVID-19 pandemic. No serious infections or cardio-/thromboembolic adverse events occurred, despite the advanced age of the patients and potential safety concerns related to JAKi [38]. One patient experienced herpes zoster. The best drug survival rate was observed in the group of patients with ≥ 2 prior bDMARD therapies (*n* = 14/18; 77.8%). In addition to the number of prior therapies, all of these patients had at least moderate disease activity at the start of the study (as measured by DAS28-CRP), fulfilling at least the first two EULAR criteria for difficult-to-treat (D2T) RA [39]. The finding that JAKi may represent a superior therapeutic strategy in D2T RA patients aligns with data from larger cohort studies [40, 41].

The study has several limitations. First, the sample size is relatively small, which may limit the generalizability of the findings. Additionally, although prospective, this is a observational study and thus lacks a control group making it difficult to compare the observed effects with other treatment methods. It should be noted that the measured changes in BMD fell within the LSC thresholds, which must be considered when interpreting the results. Furthermore, including additional markers of bone metabolism beyond AP such as P1NP or CTX would have strengthened the results.

In conclusion, baricitinib treatment was associated with stabilization of BMD loss in RA patients. However, patients who did not respond to baricitinib experienced a decline in BMD, raising the question if seen effects are achieved by controlling disease activity or if there is an additional explicit JAKi effect on bone metabolism.

## Data Availability

The data will be made available on reasonable request.
