# Correction to: Bone Mineral Density During Treatment with The Janus Kinase Inhibitor Baricitinib in Patients with Rheumatoid Arthritis: A Monocentric Observational Study

**DOI:** 10.1007/s00223-025-01451-0

**Published:** 2025-12-05

**Authors:** Nils Schulz, Thomas Asendorf, Pascal van Wijnen, Tim Wilhelmi, Ulf Müller-Ladner, Uwe Lange, Philipp Klemm

**Affiliations:** 1 Department of Rheumatology, Clinical Immunology, Osteology and Physical Medicine, Justus Liebig University Giessen, Campus Kerckhoff, Benekestr. 2-8, 61231 Bad Nauheim, Germany; 2 Department of Medical Statistics, University Medical Center Göttingen, Göttingen, Germany

**Correction to: Calcified Tissue International (2025) 116:101** 10.1007/s00223-025-01410-9

The article “Bone Mineral Density During Treatment with The Janus Kinase Inhibitor Baricitinib in Patients with Rheumatoid Arthritis: A Monocentric Observational Study”, written by “Nils Schulz1 · Thomas Asendorf2 · Pascal van Wijnen1 · Tim Wilhelmi1 · Ulf Müller-Ladner1 · Uwe Lange1 · Philipp Klemm**”**, was originally published Online First without Open Access. After publication in volume 116, issue 1, the author decided to opt for Open Choice and to make the article an Open Access publication. Therefore, the copyright of the article has been changed to © The Author(s) 2025 and the article is forthwith distributed under the terms of the Creative Commons Attribution 4.0 International License, which permits use, sharing, adaptation, distribution and reproduction in any medium or format, as long as you give appropriate credit to the original author(s) and the source, provide a link to the Creative Commons licence, and indicate if changes were made. The images or other third party material in this article are included in the article’s Creative Commons licence, unless indicated otherwise in a credit line to the material. If material is not included in the article’s Creative Commons licence and your intended use is not permitted by statutory regulation or exceeds the permitted use, you will need to obtain permission directly from the copyright holder. To view a copy of this licence, visit http://creativecommons.org/licenses/by/4.0.

